# Dietary supplementation with farnesol confers a protective effect on the intestine of broiler chickens challenged with lipopolysaccharide by reshaping intestinal flora structure and regulating TLR4/NF-κB signaling pathway

**DOI:** 10.1016/j.psj.2025.104942

**Published:** 2025-02-23

**Authors:** Peng Li, Chenyu Guo, Wenfei Tong, Shaochen Han, Xiangxue Sun, Lei Xiao, Qunbing Hu, Yongqing Hou, Binying Ding, Dan Yi

**Affiliations:** a Hubei Key Laboratory of Animal Nutrition and Feed Science, Engineering Research Center of Feed Protein Resources of Agricultural By-products, Ministry of Education,Wuhan Polytechnic University, Wuhan, Hubei 430023, PR China; b Hubei Lan Good microbial Technology Co., Ltd. Yichang, Hubei 443100, PR China; c Hubei Horwath Biotechnology Co., Ltd. Xianning, Hubei 437000, PR China

**Keywords:** Farnesol, Broiler chicken, Lipopolysaccharide, Intestinal flora, TLR4/NF-κB signaling pathway

## Abstract

Farnesol (**FAN**), one of plant essential oils, is widely found in a variety of natural plants. Studies demonstrated that FAN contributed to the antioxidant and immune function as well as improving the intestinal flora, however effects of it on the broiler chickens has not been fully characterized. In the present study, we present an undated report of its effects on growth performance, antioxidant and immune functions of broiler chickens challenged with lipopolysaccharide (**LPS**). One hundred healthy male AA^+^ broiler chickens with uniform body weight were divided into control and FAN groups, there were five replicates and 10 birds in each one. The trial lasted for 28 days, and two birds with uniform body weight were selected from each replicate to be treated with intraperitoneal injection of LPS at the end of the trial, and then samples were harvested after 3 h. Results showed that dietary supplementary with FAN tended to improve the feed conversion ratio (**FCR**) (*P* = 0.058). The levels of serum lactate dehydrogenase and IL-1β were elevated in the birds challenged with LPS, as well as the content of malondialdehyde in the ileal and liver (*P* < 0.05). Additionally, LPS treatment descended the levels of catalase and superoxide dismutase, and the ratio of villi height to crypt depth in the ileum (*P* < 0.05). Dietary supplementation with FAN was able to alleviate the abnormal changes of the above indexes caused by LPS. In addition, dietary supplementation with FAN also contributed to alleviating the up-regulation of Toll-like receptor 4 (***TLR-4***), nuclear transcription factor κB (***NF-κB***), myeloid differentiation primary response gene 88 (***MYD88***), tumor necrosis factor (***TNF-α***) and *IL-1β* transcription levels in the ileum and liver of birds challenged with LPS (*P* < 0.05). Results of intestinal flora demonstrated that the relative abundance of *Candidatus Arthromitus* was up-regulated in the ileal chyme of birds challenged with LPS, and dietary supplementation with FAN could reshape it. Intriguingly, the relative abundance of *Candidatus Arthromitus* was positively correlated with the mRNA levels of *TLR-4, NF-κB, MYD88, TNF-α* and *IL-1β* in the ileum (*P* < 0.05). In conclusion, dietary supplementation with FAN might confer a protective effect on the intestine of broiler chickens challenged with lipopolysaccharide by reshaping intestinal flora, especially *Candidatus Arthromitus,* and regulating TLR4/NF-κB signaling pathway.

## Introduction

At present, a number of countries have implemented the prohibition of antibiotics in feed, despite the improvement of antibiotic resistance and food health issues, we have to acknowledge the beneficial role of antibiotics in promoting growth and improving intestinal health in broiler chickens. An example of this is that without antibiotics in feed, it might be easier to offer an opportunity for the proliferation and colonization of *Clostridium perfringens*, pathogenic *Escherichia coli* and other pathogenic bacteria (Wickramasuriya et al., 2022). The excessive proliferation of these pathogenic bacteria would disrupt homeostasis of intestinal flora, and the intestinal barrier of birds was broken due to the harmful cellular components or metabolites of those pathogenic bacteria (Kogut et al., 2020). Lipopolysaccharide (**LPS**), also known as endotoxins, is a component of the cell wall of Gram-negative bacteria such as *Escherichia coli* and *Salmonella*. LPS has the ability to stimulate the secretion of pro-inflammatory cytokines, such as IL-1β, IL-6, and TNF-α, which opens the door to the intestinal injury of body (Lutful, 2010). Studies demonstrated that LPS induced inflammatory liver injury in chickens by activating the autophagy and necroptosis pathways (Yu et al., 2024), and LPS also caused intestinal injury in chickens by activating Toll-like receptor 4 (**TLR4**) to induce up-regulation of pro-inflammatory cytokines such as IL-6, and decreased the level of intestinal goblet cells and their expressed mucin-2 (Li et al., 2018). Previously, antibiotics in feed might have prevented these problems, in the post-antibiotic era, the development of safe and highly effective alternatives to antibiotics is one of our current priorities.

Plant essential oils are a class of volatile aromatic compounds that contributes to regulating the intestinal flora by down-regulating the number of pathogenic bacteria, and improving the intestinal health as well as growth performance of broiler chickens, which is worth paying more attention to the improvement of the economic benefits of broiler chickens (Abd et al., 2022; Zhai et al., 2018). Farnesol (FAN), one of plant essential oils, is widely present in lemongrass, tuberose, cyclamen, rose, neroli and other plants (Sell et al., 2022). Study demonstrated that FAN was a non-steroidal isoprenoid, and it could also be produced endogenously via dephosphorylation of farnesyl pyrophosphate in the cholesterol biosynthetic pathway (Costa et al., 2021). FAN has been shown to alleviate inflammation in the skin of mice or human lung adenocarcinoma H460 cells via inhibiting activation of Ras-Raf-ERK1/2 pathway and nuclear translocation of nuclear transcription factor κB (NF-κB) (Chaudhary et al., 2009; Joo and Jetten, 2008). Additionally, FAN could inhibit apoptosis by regulating the expression of B-cell lymphoma-2 associated X protein (Bax) and B-cell lymphoma/leukemia 2 (Bcl-2) proteins (Chaudhary et al., 2009), and alleviate the colon injury of rats challenged with 1,2-dimethylhydrazine by regulating the activity of cysteinyl aspartate specific proteinase 3 (Caspase 3) (Khan and Sultana, 2011). While the beneficial effects of FAN on mice or human cells have been extensively studied, effects of FAN on the broiler chickens have not been fully characterized.

Currently, the construction of intestinal injury models by intraperitoneal injection of LPS has been widely recognized (Chen and Yu, 2021; Wang et al., 2021). We hold it might be interesting to investigate the protective effect of dietary supplementary with FAN on growth performance and intestinal health in broiler chickens based on this LPS challenge model. Additionally, we also tried to study the protective mechanism of FAN on intestinal health from the changes of intestinal flora structure. Our research might provide a solid theoretical basis for developing alternatives to antibiotics in the feed of broiler chickens.

## Materials and methods

### Animal and diet

The animal study was carried out in the Wuhan Polytechnic University (Hubei, China), and the experimental design scheme was approved by the Animal Welfare Ethics Committee of the unit. The approved animal welfare number is WPU202307008. One hundred healthy 1-day-old, male AA^+^ broiler chickens with uniform weight were randomly assigned into two treatment groups. Five replicates in each group and ten birds in each replicates. Birds in the control group were fed with the basic diet, and the basal diet and nutrient levels arranged in Table 1 were designed according to nutritional requirements criteria for white feather broilers about Ministry of Agriculture of the People's Republic of China, 2004. Birds in the FAN group were fed the basic diet supplemented with 500 mg/kg FAN, purchased from Hubei Xinghengye Technology Co., LTD., had a purity of more than 96 %, which was detected by high performance liquid chromatography. During the animal trial, all birds raised in wire cages with the size of 1.2 m × 1.2 m got free access to water and feed, a 24 h light regime was implemented. For the first four days of the trial, the room temperature was controlled at 35 °C. After that, the room temperature was reduced by 1 °C per day until it was stabilized at 25 °C. The animal trial lasted for 28 days, at the end of the trial, four chickens were selected at random from each replicated group, two of birds were intraperitoneal injected of LPS (*E. coli* serotype 055: B5; Sigma Chemical Inc., St. Louis, MO, USA) with does of 1 mg/kg body weight (Zhang et al., 2024), and another two were treated with the same volume of normal saline. 3 h later, blood was collected from the wing vein, and slaughtered for harvesting the thymus, spleen, bursa of fabricius, cecal tonsil, abdominal fat and liver, and the relative weight of the tissues was described in terms of the ratio of the weight of the tissues to the body weight of the broiler, which was expressed in g/kg. Additionally, the ileum, ileal chyme and liver were harvested for detection analysis. To be clear, the growth performance from day 1 to day 28 of the animal trial was analyzed between control and FAN group. Briefly, birds that had fasted for 8 h on days 1, 14, and 28 were weighed and the remaining feed weight was recorded. The feed weight at the start of the trial was recorded, hence total feed intake and total weight gain from days 1 to 14, from days 1 to 28, and from days 14 to 28 could be obtained, and ADG and ADFI could be calculated accordingly. FCR was characterized by the ratio of ADFI to ADG. And the subsequent analysis indexes were designed with 2 × 2 factor, and the four treatment groups were divided into Control, FAN group, LPS and FAN+ LPS group.

**Table 1:** The feed ingredient composition and nutrient levels, on an air-dried basis.

Items	Starter period
**(%, Unless Otherwise Indicated)**	**(d1 to d28)**
**Ingredient**	
Corn	51.39
Soybean meal	38
Corn gluten meal	3
Soybean oil	3.4
Dicalcium phosphate	1.9
Limestone	1.2
Sodium chloride	0.35
DL-methionine	0.26
Choline chloride	0.25
Trance mineral premix 1	0.2
Vitamin premix 2	0.05
Nutrient levels 3	
Crude protein	23.49
ME (MJ/kg)	14.77
Ca	1.15
Available P	0.6
Lysine	1.32
Methionine + cystine	0.93

1 The trace mineral premix supplied the following per kilogram of diet: copper, 8 mg; iron, 80 mg; zinc, 75 mg; manganese, 100 mg; selenium, 0.15 mg; iodine, 0.35 mg.

2 The vitamin premix supplied the following per kilogram of diet: vitamin A, 8,000 IU; vitamin D, 32,50 IU; vitamin E, 30 IU; vitamin K, 32.65 mg; vitamin B1, 2 mg; vitamin B2, 6 mg; vitamin B6, 5 mg; vitamin B12, 0.025 mg; biotin, 0.0325 mg; folic acid, 1.25 mg; pantothenic acid, 12 mg; nicotinic acid, 50 mg.

3 Nutrient levels was a calculated value.

### Blood biochemical index and antioxidant-related parameters in ileum and liver

Blood was collected from the subwing vein, and the serum was obtained by centrifuging the blood at 3,000 r/min and 4 °C for 10 min and was stored at −80 °C for later use. The levels of total bilirubin (**TB**), glutamic oxalacetic transaminase (**AST**), Glutamyltransferase (**GGT**), alkaline phosphatase (**ALP**), total cholesterol (**TC**), triglycerides (**TG**), high-density lipoprotein (**HDL**), low-density lipoprotein (**LDL**) and lactate dehydrogenase (**LDH**) in the serum were measured using an automated biochemical analyzer (HITACHI 7020, Japan). The kits of above blood biochemical index were purchased from Shanghai Kehua Biological Engineering Co., LTD, China.

Ileum and liver were harvested at the end of the trial, and then those samples were frozen in liquid nitrogen and ground to a powder. According to the principle of 1: 9 weight to volume ratio, those samples were evenly mixed with pre-cooled normal saline. The mixture was placed on the vortex meter and thoroughly mixed, and centrifuged at 3,500 r/min and 4 °C for 10 min to separate the supernatant for later use. The contents of catalase (**CAT**), total superoxide dismutase (**T-SOD**), H2O2 and malondialdehyde (**MDA**) in the ileum, Glutathione peroxidase (**GSH-Px**), Myeloperoxidase (**MPO**), T-SOD and MDA in the liver were measured following the protocol of kits purchased from Nanjing Jiancheng Institute of Biological Engineering, Jiangsu, China.

### Ileal morphological structure and the level of serum immune factors

At the end of the trial, about 1 cm ileal tube was collected and the chyme was rinsed with room temperature normal saline and placed in a 4 % paraformaldehyde solution. The ileal tissues were buried in wax blocks, sliced by cryotome and stained with eosin and hematoxylin. In each ileal section, 10 morphologically intact intestinal villi were selected and a thousand-screen high-definition color pathology graphic analysis system in the Olymps BX-41TF microscope was used to investigate the villi height (**VH**), villus surface area (**VSA**) and crypt deapth (**CD**), and then the ratio of VH to CD was calculated.

The serum was harvested according to the protocol as described above, the kit purchased from Nanjing Jiancheng Institute of Biological Engineering, Jiangsu, China was used to measure the level of lysozyme in the serum. The ELISA kits purchased from Beijing Solarbio Science & Technology Co.,Ltd, Beijing, China were used to investigate the contents of interleukin 1β (**IL-1β**) and transforming growth factor β (**TGF-β**).

### Transcription level of genes in the ileum and liver

At the end of the trial, ileum and liver were harvested and frozen in liquid nitrogen and ground to a powder. The total RNA was obtained with the Trizol reagent (Takara, Dalian, China), and a Nano Drop 2000 spectrophotometer (Thermo Scientific, USA) was used to measure the concentration of RNA. To ensure the RNA purity, the ratio of OD260 to OD 280 should be approximately 2.0. Then, cDNA was prepared following the protocol of the gDNA Eraser (Takara, Dalian, China), and the real-time PCR reaction was performed on an applied biosystems 7500 fast Real-time PCR system (Foster City, CA). The real-time PCR reaction program was described as 95 °C for 30 s at an initial denaturation step, and then 40 cycles at 95 °C for 5 s was required. Finally, 60 °C for 34 s at an annealing and extension step was performed. The protocol of 2^−ΔΔCt^ method described in the previous study (Fu et al., 2010) was used to analyze the relative expression of each gene, and *β-actin* was executed as the reference gene during data analysis. The primer sequences of all the genes tested in the present study were arranged in Table 2.

**Table 2:** The primer sequences of genes 1.

Gene name	Primer sequence (5′ to 3′)		Accession number
*β-actin*	Forward	ACTCTGGTGATGGTGTTAC	NM 205518
	Reverse	GGCTGTGATCTCCTTCTG	
*Claudin-1*	Forward	CATACTCCTGGGTCTGGTTGGT	AY750897
	Reverse	GACAGCCATCCGCATCTTCT	
*ZO-1*	Forward	CTTCAGGTGTTTCTCTTCCTCCTC	XM_413773
	Reverse	CTGTGGTTTCATGGCTGGATC	
*Mucin-2*	Forward	TTCATGATGCCTGCTCTTGTG	XM_421035
	Reverse	CCTGAGCCTTGGTACATTCTTGT	
*TLR-4*	Forward	AGTCTGAAATTGCTGAGCTCAAAT	NM_001030693.1
	Reverse	GCGACGTTAAGCCATGGAAG	
*MYD88*	Forward	TGCAAGACCATGAAGAACGA	NM_001030962.4
	Reverse	TCACGGCAGCAAGAGAGATT	
*NF-κB*	Forward	GTGTGAAGAAACGGGAACTG	NM_205129.1
	Reverse	GGCACGGTTGTCATAGATGG	
*IL-1β*	Forward	ACTGGGCATCAAGGGCTA	XM_015297469.2
	Reverse	GGTAGAAGATGAAGCGGGTC	
*TNF-α*	Forward	GAGCGTTGACTTGGCTGTC	XM_040647309.1
	Reverse	AAGCAACAACCAGCTATGCAC	
*TGF-β*	Forward	TCATCACCAGGACAGCGTTA	NM_001031045.3
	Reverse	TGTGATGGAGCCATTCATGT	

1 Primers designed using Primer Express software (Sangon Biotech, Shanghai, China).

### 16 s sequencing of bacteria in ileal chyme

At the end of the trial, the ileal chyme was harvested, and the genomic DNA of bacteria in the ileal chyme was collected following the protocol of QIAamp fast DNA stool mini kit (Qiagen, Hilden, Germany). And then, the concentration of DNA was measured by a Nano Drop 2000 spectrophotometer (Thermo Scientific, USA) for the on-machine sequencing in HiSeq2500 PE250 and analysis. The V3-V4 region universal primers named 338 F (5′-ACTCCTACGGGAGGCAGCA-3′) and 806 R (5′-GGACTACHVGGGTWTCTAAT-3′) of 16S rDNA were used to amplify bacterial DNA, the PCR amplified products were purified, the concentration and homogenization were determined, and further sequencing libraries were constructed according to the previous study (Li et al., 2022). On-machine sequencing was carried out and the analysis was completed by Beijing Nuohe Zhiyuan Bio-Information Technology Co., Ltd. Qiime software (Qiime2-2019.7, Nature Biotechnology) was used to generate species abundance tables in genus levels. R (Version 3.0.3) was used to get the Venn diagram and PCOA, and LEfSe software (Version 1.0) was used to obtain the LEFse results which strictly implement the standard of LDA scores greater than 4.

### Statistical analysis

The conventional linear model program in SPSS 23.0 software (SPSS Inc., Chicago, IL) was used to analyze the data, a 2 × 2 factor arrangement to analyze the effects of LPS and FAN, as well as the interaction of these two factors. The One-way ANOVA and Duncan's multiple comparisons were used in SPSS 23.0 software (SPSS Inc., Chicago, IL) to analyze the data of α-diversity about intestinal flora in the ileal chyme, and the independent sample T-test was used to analyze the growth performance between CTR and FAN group. Pearson correlation coefficient in SPSS 23.0 software (SPSS Inc., Chicago, IL) was used to investigate the correlation between the measured indicators and the relative abundance of representative bacteria, and the date was presented graphically by the Graphpad Prism 8.0 software. In the results, *P* < 0.05 was considered to be significant, 0.05< *P* < 0.1 indicated a trend of significant differences between groups additionally.

## Results

### Growth performance, blood biochemical index and antioxidant-related parameters in ileum and liver

Dietary supplementary with FAN had no effect on ADG and ADFI, however FAN tended to improve the feed conversion ratio (FCR) from day 1 to day 14 of the trial (*P* = 0.058) (Table 3). Dietary supplementary with FAN did not affect the organ mass index of birds, and interestingly, it reduced the level of abdominal fat in broiler chickens (*P* < 0.05) (Table 4). The levels of TB, TC and HDL were descended, and the contents of TG and LDH in the serum were elevated with LPS challenged, dietary supplemetary with FAN alleviated the down-regulation of serum HDL in the birds challenged with LPS, and down-regulated the level of LDH in the serum (Table 5).

**Table 3:** Growth performance.

Item		CTR	FAN	*P*-value
BW, g	d1	40.05±0.32	40.01±0.18	0.824
	d14	361.62±13.95	364.46±9.46	0.716
	d28	1265.2 ± 71.08	1248.24±56.41	0.687
d1-d14	ADG, g	24.74±1.07	24.96±0.72	0.712
	ADFI, g	32.64±1.55	31.37±1.92	0.285
	FCR	1.32±0.04	1.26±0.05	0.058
d14-d28	ADG, g	64.54±4.13	63.13±4.36	0.613
	ADFI, g	101.74±9.96	97.33±6.92	0.440
	FCR	1.57±0.07	1.54±0.04	0.419
d1-d28	ADG, g	45.38±2.63	44.75±2.09	0.688
	ADFI, g	68.47±5.83	65.57±3.28	0.362
	FCR	1.51±0.05	1.47±0.03	0.154

ADFI = average daily feed intake, ADG= average daily gain, FCR = feed conversion ratio, BW = body weight.

**Table 4:** Parameters of tissue weight relative to broiler body weight, g/kg.

Item^1^		Thymus	Spleen	Bursa of fabricius	Cecal tonsil	Abdominal fat	Liver
FAN	LPS						
-	-	1.89	0.99	2.42	0.38	6.97	19.35
-	+	1.81	0.95	2.15	0.40	7.27	18.89
+	-	1.64	0.88	2.37	0.43	5.55	18.29
+	+	1.69	0.82	2.36	0.37	4.91	18.70
SEM^2^		0.055	0.045	0.101	0.013	0.278	0.238
Main effect							
FAN	-	1.85	0.97	2.29	0.39	7.12^A^	19.12
	+	1.67	0.85	2.37	0.40	5.23^B^	18.50
LPS	-	1.76	0.93	2.40	0.40	6.26	18.82
	+	1.75	0.88	2.25	0.38	6.09	18.80
Interaction, *P*-value							
FAN		0.120	0.194	0.697	0.742	0.003	0.205
LPS		0.927	0.588	0.483	0.460	0.763	0.965
FAN*LPS		0.559	0.885	0.528	0.112	0.408	0.368

^A,B^ Means in the same column without common superscripts differ significantly (*P* < 0.05), same shoulder letters indicate no difference (*P* > 0.1), the same below.

1 (-) Means not added, (+) means to add.

2 SEM, standard error of the mean.

**Table 5:** Effects of serum biochemical parameters.

Item		TB, μmol/L	AST,U/L	ALP, U/L	GGT, U/L	TC, mmol/L	TG, mmol/L	HDL, mmol/L	LDL, mmol/L	LDH, U/L
FAN	LPS									
-	-	7.65	243.50	1651.68	18.50	3.22^a^	0.33	2.21^a^	0.50	1032.58
-	+	5.78	310.00	1590.00	20.00	2.37^b^	0.39	1.53^c^	0.38	1308.15
+	-	7.85	258.20	1790.20	20.80	2.98^a^	0.33	2.07^a^	0.45	886.46
+	+	7.09	260.33	1735.67	21.00	2.64^b^	0.44	1.88^b^	0.45	953.01
SEM		0.208	10.726	77.441	1.103	0.064	0.012	0.055	0.021	38.479
Main effect										
FAN	-	6.71	276.75	1620.84	19.25	2.79	0.36	1.87	0.44	1170.36^A^
	+	7.47	259.27	1762.93	20.90	2.81	0.39	1.97	0.45	919.74^B^
LPS	-	7.75^A^	250.85	1720.94	19.65	3.10^A^	0.33^B^	2.14^A^	0.48	959.52^B^
	+	6.43^B^	285.17	1662.83	20.50	2.51^B^	0.41^A^	1.70^B^	0.42	1130.58^A^
Interaction ,P-value										
FAN		0.098	0.434	0.381	0.472	0.521	0.284	0.344	0.820	0.009
LPS		0.010	0.141	0.715	0.708	0.002	0.010	0.003	0.166	0.050
FAN*LPS		0.209	0.164	0.982	0.774	0.030	0.309	0.050	0.179	0.204

^a,b,c^ Means in the same column without common superscripts differ significantly between four group, and ^A,B^ Means in the same column without common superscripts differ significantly about the main effect of FAN or LPS (*P* < 0.05), same shoulder letters indicate no difference (*P* > 0.1), the same below.

^1^ (-) Means not added, (+) means to add.

^2^ SEM, standard error of the mean.

The levels of CAT and T-SOD were descended and MDA was raised in the ileum of birds challenged with LPS, dietary supplemetary with FAN raised the level of ileal CAT and was able to alleviating the up-regulation of ileal MDA in the birds challenged with LPS (*P* < 0.05) (Table 6). Additionally, the level of GSH-Px was descended and MDA was elevated in the liver of birds challenged by LPS, dietary supplemetary with FAN reduced the level of MPO in the birds (*P* < 0.05) (Table 6).

**Table 6:** Results of antioxidant-related parameters in ileum and liver.

Item		Ileum				liver			
FAN	LPS	CAT	H_2_O_2_	MDA	SOD	GSH-Px	MDA	MPO	SOD
-	-	3.64	9.26	1.75^c^	379.97	154.20	3.45^a^	0.29	148.20
-	+	0.52	9.04	5.50^a^	282.89	106.88	4.08^a^	0.32	118.48
+	-	4.93	7.05	2.21^c^	413.32	167.91	1.88^b^	0.13	177.28
+	+	1.17	7.51	4.35^b^	351.57	104.57	5.07^a^	0.19	158.55
SEM		0.143	0.759	0.155	13.338	10.081	0.252	0.020	9.323
Main effect									
FAN	-	2.08^B^	9.15	3.63	331.43	130.54	3.77	0.30^A^	133.34
	+	3.05^A^	7.28	3.28	382.44	136.24	3.48	0.16^B^	167.92
LPS	-	4.28^A^	8.15	1.98^B^	396.65^A^	161.05^A^	2.67^B^	0.21	162.74
	+	0.85^B^	8.28	4.93^A^	317.23^B^	105.73^B^	4.58^A^	0.25	138.52
Interaction, P-value									
FAN		0.008	0.248	0.288	0.088	0.784	0.581	0.007	0.097
LPS		<0.001	0.938	<0.001	0.016	0.023	0.004	0.325	0.226
FAN*LPS		0.293	0.827	0.028	0.524	0.700	0.031	0.763	0.775

^a,b,c^ Means in the same column without common superscripts differ significantly between four group, and ^A,B^ Means in the same column without common superscripts differ significantly about the main effect of FAN or LPS (*P* < 0.05), same shoulder letters indicate no difference (*P* > 0.1), the same below.

^1^ (-) Means not added, (+) means to add.

^2^ SEM, standard error of the mean.

### Ileal morphological structure and the level of serum immune factors

Dietary supplemetary with FAN increased the ileal villus surface area (*P* < 0.05) (Table 7), but it had no effects on the villi height and crypt depth of ileum. The crypt depth was raised and the ratio of villi height to crypt depth of ileum was descended with LPS (*P* < 0.05) (Table 7), dietary supplemetary with FAN failed to alleviate it. In addition, the levels of TGF-β, IL-1β and lysozyme in the serum were elevated of the birds challenged with LPS, dietary supplemetary with FAN helped to alleviate the up-regulation of TGF-β and lysozyme in serum of birds challenged with LPS (*P* < 0.05) (Table 8).

**Table 7:** Morphological parameters of ileum.

Item		VH, μm	CD, μm	VSA, μm^2^	VH/CD
FAN	LPS				
-	-	758.75	112.49	89123.45^b^	6.89
-	+	769.67	132.42	91901.90^b^	6.08
+	-	848.35	108.36	107663.82^a^	7.91
+	+	761.15	122.42	88576.94^b^	6.40
SEM		13.987	4.048	2346.103	0.221
Main effect					
FAN	-	764.21	122.46	90512.68	6.49
	+	804.75	115.39	98120.38	7.16
LPS	-	803.55	110.42^B^	98393.64	7.40^A^
	+	765.41	127.42^A^	90239.42	6.24^B^
Interaction, *P*-value					
FAN		0.158	0.390	0.116	0.140
LPS		0.184	0.045	0.093	0.014
FAN*LPS		0.090	0.719	0.027	0.439

^a,b,c^ Means in the same column without common superscripts differ significantly between four group, and ^A,B^ Means in the same column without common superscripts differ significantly about the main effect of FAN or LPS (*P* < 0.05), same shoulder letters indicate no difference (*P* > 0.1), the same below.

^1^ (-) Means not added, (+) means to add.

^2^ SEM, standard error of the mean.

**Table 8:** Levels of immune factors in the serum.

Item		TGF-β	IL-1β	lysozyme
FAN	LPS			
-	-	200.13^c^	177.14	2.79^c^
-	+	339.52^a^	309.46	4.46^a^
+	-	275.65^b^	164.96	3.99^ab^
+	+	232.19^bc^	239.83	3.61^b^
SEM		12.100	10.828	0.093
Main effect				
FAN	-	269.83	243.30	3.62
	+	253.92	202.40	3.80
LPS	-	237.89	171.05^B^	3.39^B^
	+	285.86	274.65^A^	4.04^A^
Interaction, *P*-value				
FAN		0.520	0.077	0.351
LPS		0.065	< 0.001	0.003
FAN*LPS		0.002	0.203	< 0.001

^a,b,c^ Means in the same column without common superscripts differ significantly between four group, and ^A,B^ Means in the same column without common superscripts differ significantly about the main effect of FAN or LPS (*P* < 0.05), same shoulder letters indicate no difference (*P* > 0.1), the same below.

^1^ (-) Means not added, (+) means to add.

^2^ SEM, standard error of the mean.

### Transcription level of genes in the ileum and liver

Compared with the control group, the mRNA level of ileal *Mucin-2* was down-regulated in the birds challenged with LPS, dietary supplemetary with FAN contributed to alleviating it and up-regulated the mRNA levels of ileal *Claudin-1* and *ZO-1* (*P* < 0.05) (Table 9). It was worth mentioning that LPS challenge up-regulated the transcription levels of *TLR-4, MYD88, NF-κB, TNF-α, TGF-β* and *IL-1β* in the ileum, intriguingly, dietary supplemetary with FAN helped to alleviate it (*P* < 0.05) (Table 9).

**Table 9:** Genes mRNA levels in the ileum.

Item		*Claudin-1*	*ZO-1*	*Mucin-2*	*TLR-4*	*MYD88*	*NF-κB*	*IL-1β*	*TNF-α*	*TGF-β*
FAN	LPS									
-	-	1.00^bc^	1.00^b^	1.00^b^	1.00^c^	1.00^b^	1.00^b^	1.00^c^	1.00^c^	1.00^d^
-	+	0.81^c^	0.70^b^	0.62^c^	2.37^a^	1.69^a^	2.05^a^	2.90^a^	2.06^a^	2.29^b^
+	-	1.28^b^	0.91^b^	0.83^b^	1.45^b^	1.32^b^	1.37^b^	0.94^c^	1.38^b^	3.01^a^
+	+	1.90^a^	2.86^a^	2.33^a^	1.50^b^	1.23^b^	1.23^b^	1.83^b^	1.38^b^	1.52^c^
SEM		0.075	0.058	0.03	0.054	0.063	0.069	0.092	0.062	0.06
Main effect										
FAN	-	0.91^B^	0.85^B^	0.81^B^	1.69	1.35	1.53	1.95^A^	1.53	1.65^B^
	+	1.59^A^	1.88^A^	1.58^A^	1.47	1.27	1.30	1.39^B^	1.38	2.27^A^
LPS	-	1.15	0.95^B^	0.91^B^	1.22^B^	1.16^B^	1.18^B^	0.97^B^	1.19^B^	2.01
	+	1.35	1.78^A^	1.48^A^	1.94^A^	1.46^A^	1.64^A^	2.37^A^	1.72^A^	1.91
Interaction, *P*-value										
FAN		<0.001	<0.001	<0.001	0.060	0.580	0.116	0.006	0.249	<0.001
LPS		0.183	<0.001	<0.001	<0.001	0.027	0.004	<0.001	<0.001	0.421
FAN*LPS		0.012	<0.001	<0.001	<0.001	0.005	<0.001	0.012	<0.001	<0.001

^a,b,c^ Means in the same column without common superscripts differ significantly between four group, and ^A,B^ Means in the same column without common superscripts differ significantly about the main effect of FAN or LPS (*P* < 0.05), same shoulder letters indicate no difference (*P* > 0.1), the same below.

^1^ (-) Means not added, (+) means to add.

^2^ SEM, standard error of the mean.

In the liver, similar outcomes showed that the mRNA levels of *TLR-4, MYD88, NF-κB, TNF-α, TGF-β* and *IL-1β* were up-regulated in liver of birds challenged with LPS. dietary supplemetary with FAN alleviated the up-regulation of *TLR-4, MYD88, NF-κB, TNF-α* and *IL-1β* in the liver of birds challenged with LPS, and further up-regulated the transcription level of *TGF-β* (*P* < 0.05) (Table 10).

**Table 10:** Genes mRNA levels in the liver.

Item		*TLR-4*	*MYD88*	*NF-κB*	*IL-1β*	*TNF-α*	*TGF-β*
FAN	LPS						
-	-	1.00^c^	1.00^c^	1.00^c^	1.00^c^	1.00^c^	1.00^d^
-	+	3.47^a^	4.71^a^	2.63^a^	56.09^a^	19.90^a^	2.48^b^
+	-	1.58^b^	1.49^c^	1.34^c^	1.85^c^	1.90^c^	1.57^c^
+	+	1.44^b^	3.49^b^	2.01^b^	43.60^b^	5.69^b^	4.82^a^
SEM		0.092	0.136	0.073	1.259	0.573	0.085
Main effect							
FAN	-	2.23^A^	2.85	1.81	28.54^A^	10.45^A^	1.74^B^
	+	1.51^B^	2.49	1.67	22.73^B^	3.79^B^	3.19^A^
LPS	-	1.29^B^	1.25^B^	1.17^B^	1.43^B^	1.45^B^	1.28^B^
	+	2.45^A^	4.10^A^	2.32^A^	49.84^A^	12.79^A^	3.65^A^
Interaction, *P*-value							
FAN		0.001	0.196	0.346	0.031	<0.001	<0.001
LPS		<0.001	<0.001	<0.001	<0.001	<0.001	<0.001
FAN*LPS		<0.001	0.005	0.004	0.015	<0.001	<0.001

^a, b, c^ Means in the same column without common superscripts differ significantly between four group, and ^A,B^ Means in the same column without common superscripts differ significantly about the main effect of FAN or LPS (*P* < 0.05), same shoulder letters indicate no difference (*P* > 0.1), the same below.

^1^ (-) Means not added, (+) means to add.

^2^ SEM, standard error of the mean.

### 16 s sequencing of bacteria in ileal chyme and correlation analysis

**Fig. 1:**
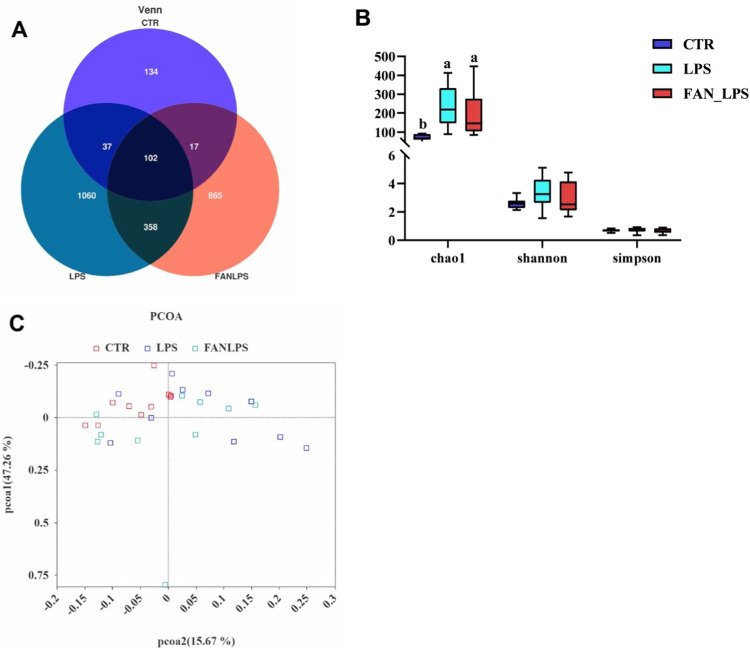
Effects of FAN on the ileal flora in the birds challenged with LPS. Among them, the Venn and PCOA diagram were arranged in Fig. 1A and 1C, the outcomes of α-diversity in the ileal flora were showed in Fig. 1B. In Fig. 1A, each circle in the Venn diagram represented a group of samples, and the number of overlapping circles represented the number of operational taxonomic units (OTUS) common among the groups, while the number without overlapping parts represented the number of unique OTUs of the group. In Fig. 1B, different letters of the column indicated significant differences (*P* < 0.05).

**Fig. 2:**
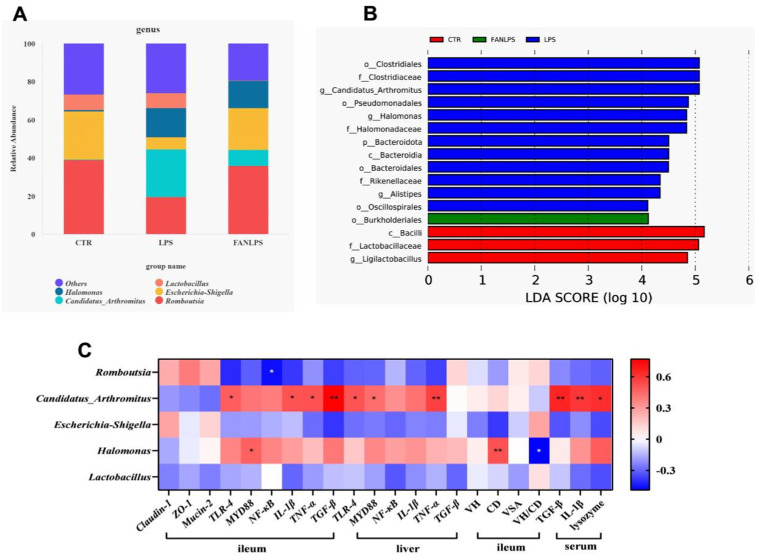
The analysis results of different bacteria in intestinal flora at genus level, the outcomes of LEFse analysis and the correlation analysis of key bacteria and intestinal physiological function. The results of intestinal flora at genus level and LEFse analysis were arranged in Fig. 2A and 2B, the outcomes of the correlation analysis of key bacteria and intestinal physiological function was showed in Fig. 2C, among them, * means 0.01< *P* < 0.05, **means 0.001 < *P* < 0.01, and the red color indicate a strong positive correlation (correlation coefficient = 1), while the purple color indicates a strong negative correlation (correlation coefficient = −1).

The number of OTUs shared by the control, LPS and FAN-LPS groups was 102, there was 134 unique OTUs in the control group, 1060 in the LPS group and 865 in the FAN-LPS group (Fig. 1A). LPS challenge elevated the chao 1 index (*P* < 0.05), and the ileal flora structure was different with or without LPS treatment (Fig. 1B, C). Specifically, the relative abundance of *Romboutsia* and *Escherichia-shigella* were down-regulated, it of *Candidatus-Arthromitus* and *Halomonas* were up-regulated in the ileal chyme of birds challenged with LPS, dietary supplemetary with FAN contributed to reshaping it (Fig. 2A).

The LEFse results showed that the dominant bacterial population in LPS group were *Clostridiales, Candidatus-Arthromitus* and *Halomonas.* The dominant bacterial population in FAN-LPS group was *Burkholderiales* (Fig. 2B). The outcomes of correlation analysis demonstrated that the relative abundance of *Romboutsia* was negatively correlated with the transcription levels of ileal *NF-κB* (*P* < 0.05)*.* Interestingly, the mRNA levels of ileal *TLR-4, MYD88, NF-κB, TNF-α, TGF-β* and *IL-1β* were positively correlated with the relative abundance of *Candidatus-Arthromitus*. Additionally, the relative abundance of *Halomonas* was positively correlated with ileal crypt depth and the transcription levels of ileal *MYD88*, and negatively correlated with the ratio of villi height to crypt depth in the ileum (*P* < 0.05) (Fig. 2C).

## Discussion

Natural plant active ingredients are considered to have great potential to replace antibiotics in feed. This is attributed to its beneficial effects on growth performance and intestinal health. Among them, plant essential oils have attracted a lot of attention in broiler production. To illustrate, a study demonstrated that dietary supplementary with essential oils of thyme and rosemary elevated the body weight and improve the feed conversion ratio (Mueller et al., 2012). Another study found that dietary supplementary with essential oils of oregano helped to improve the growth performance and immunity (Su et al., 2021). However, the effect of FAN, an acyclic sesquiterpene alcohol present in essential oils of plants, on broiler production have not been fully characterized. In the present study, we present an undated report of dietary supplementary with FAN improve the feed conversion ratio during the early stage of animal trial. A possible explanation was that the physiological function of the intestine in young animals might not be fully developed, and it was easily stimulated by harmful factors in the environment or diet. Considering the effects of FAN on immune and antioxidant function (Balaraman et al., 2021; Sell et al., 2022), we believed that FAN might shape a healthy intestine for broilers, which helps birds to be lower external stimuli and thus improve growth performance. To further explore this concept we investigated the physiology of the intestine in the birds challenged with LPS.

Study demonstrated that LPS challenge caused immune homeostasis imbalance and physiological dysfunction (Mizobuchi and Soma, 2021). This was similar to what we observed in the present study that the levels of TB, TC and HDL were abnormally variable with LPS challenge. Diet FAN reversed the HDL changes described above, which illuminated that FAN helped maintain physiological homeostasis under stress. Additionally, FAN dropped the abdominal fat of birds, it might be related to the regulation of HDL level by FAN, the detailed mechanism here deserved further investigation. A study found that the serum lactate dehydrogenase would be elevated in the condition of intestinal injury (Luo et al., 2023). In our present study, LPS challenge raised the serum lactate dehydrogenase and dietary supplementary with FAN was able to alleviate it, suggesting that FAN might contribute to repairing intestinal injury caused by LPS. To gain more insight, we conducted the following study.

The imbalance of intestinal oxidation and antioxidant homeostasis might result in the mass production of oxygen free radicals or peroxide products, for instance MDA and reactive oxygen species (Wen et al., 2023). In the present study, LPS raised the level of MDA in the ileum and liver, it suggested that LPS caused oxidative stress injury, this was basically consistent with the previous study (Yi et al., 2014; Li et al., 2024). Under oxidative stress, the antioxidant system was activated and some antioxidant enzymes, such as SOD, CAT and GSH-Px were expressed to remove peroxide products (Liu et al., 2021). In the present study, diet supplemented with FAN elevated the level of ileal CAT and descended the content of MDA in the ileum and liver of birds challenged with LPS, those outcomes demonstrated that FAN contributed to improving oxidative stress and antioxidant homeostasis imbalance induced by LPS in birds.

We further investigated intestinal barrier and immune function and found that dietary supplementary with FAN elevated the villi surface area, while FAN failed to alleviate the deterioration of villi morphology and structure caused by LPS. Mucin was attached to the intestinal villi as the first barrier of the intestine, which could resist the stimulation of the intestine by external stimuli (Breugelmans et al., 2022). The intestinal binding proteins, such as claudin-1 and ZO-1, were also the molecular basis of the intestinal barrier (Kuo et al., 2022). Our results showed that dietary supplementary with FAN inhbited the down-regulation of mRNA level about ileal *Mucin-2* in birds challenged with LPS, indicating that FAN may strengthen intestinal barrier function by regulating the mucin-2 expression. Study demonstrated that TLR4/NF-κB signaling pathway was activated by LPS, and some inflammatory cytokines were over-expressed, which further aggravated tissue injury (Tang et al., 2021). In the present study, we investigated it and the consistent results were observed. It should be pointed out that dietary supplementary with FAN could inhibit the activation of TLR4/NF-κB signaling pathway and alleviate the expression of inflammatory cytokines induced by LPS. Which was consistent not only in the ileum, but also in the liver. Our study revealed that FAN might alleviate the tissue injury caused by LPS via regulating TLR4/NF-κB signaling pathway.

A multitude of studies have confirmed that the way in which natural plant active ingredients exert their biological effects may depend on the intestinal flora (Xu et al., 2017). Hence, we tried to investigate above observations based on this, and found FAN helped to alleviate the structural dysregulation of ileal flora in birds induced by LPS stimulation. Among them, some characteristic bacteria attracted our attention. Specifically, we observed that LPS stimulation caused down-regulation of *Romboutsia* and up-regulation of *Candidatus-Arthromitus*. Our previous studies have repeatedly confirmed that *Romboutsia* plays an important role in regulating intestinal immune function and maintaining intestinal health (Li et al., 2022, 2023). Song et al. demonstrated that *Candidatus-Arthromitus* was also supposed to be the key role in maintaining intestinal immune homeostasis (Song et al., 2023). To gain more insight about it, we analyzed the correlation between the relative abundance of *Romboutsia* as well as *Candidatus-Arthromitus* and the intestinal barrier and immune-related indicators investigated in this study. Interestingly, most of the immune-related indicators were highly correlated with these two bacteria. Beyond that, our outcomes suggested that *Halomonas* might have great potential to regulate intestinal health, which was basically consistent with the previous study (Yu et al., 2021).

Our outcomes enlightened us that dietary supplementation with FAN conferred a protective effect on the intestine of birds challenged with LPS, it might be one of the reasons why FAN improved the feed conversion ratio for birds. The mechanism here might be that FAN reshaped the structure of intestinal flora and regulated the TLR4/NF-κB signaling pathway. Although a detailed investigation of mechanism of FAN regulating intestinal health was beyond the scope of this work, we acknowledged that dietary supplementary with FAN offered exciting opportunities to the growth performance and intestinal health.

## Conclusion

Dietary supplementation with farnesol confers a protective effect on the intestine of broiler chickens challenged with lipopolysaccharide by reshaping intestinal flora structure and regulating TLR4/NF-κB signaling pathway.

## Author contributions

Peng li designed the study, wrote the original draft and for funding acquisition. Chenyu Guo, Wenfei Tong, Shaochen Han, Xiangxue Sun, Lei Xiao, Qunbing Hu for the data curation, investigation and formal analysis. Dan Yi, Bingying Ding and Yongqing Hou participated in writing, reviewing and editing. All authors contributed to the data interpretation and approved the final version of the manuscript.

## Declaration of competing interest

The authors declared that they have no conflicts of interest to this work. We declare that we do not have any commercial or associative interest that represents a conflict of interest in connection with the work submitted.
